# Vitamin E acetate and its aerosol activate aryl hydrocarbon receptor signaling and exacerbate inflammation in human U937-derived macrophages

**DOI:** 10.1016/j.tiv.2026.106196

**Published:** 2026-01-08

**Authors:** Xiaohan Li, Andrea Rossi, Thomas Haarmann-Stemmann, Elliot R. Spindel, Jack C. Connolly, Allison K. Ehrlich, Kent E. Pinkerton, Christoph F.A. Vogel

**Affiliations:** a Center for Health and the Environment, University of California, Davis, CA, USA; b Leibniz Research Institute for Environmental Medicine, 40225 Düsseldorf, Germany; c Oregon Health Sciences University, Portland, OR, USA; d Department of Pediatrics, School of Medicine, University of California, Davis, CA, USA; e Department of Environmental Toxicology, University of California, Davis, CA, USA

**Keywords:** AhR, COX-2, EVALI, IL-8, Inflammation, Oxidative stress, VEA

## Abstract

In 2019, a number of patients were hospitalized after the use of electronic cigarettes and displayed acute lung injuries. Such injury was categorized as e-cigarette or vaping associated lung injury (EVALI). Among these patients, Vitamin E acetate (VEA) was detected in most used electronic cigarette cartridges as well as the patients’ bronchoalveolar lavage fluid, suggesting VEA to be a culprit of causing lung injury. Although further experiments verified the potential of VEA aerosol to cause cytotoxicity and lung injury, mechanisms of VEA aerosol toxicity are not well understood. In this study, we tested the toxicity of VEA, and its aerosol using a human macrophage model. VEA aerosols significantly induced oxidative stress as well as proinflammatory responses. In addition, the aerosol activated the aryl hydrocarbon receptor (AhR) signaling pathway, inducing CYP1A1 expression in human U937 monocyte-derived macrophages. Additionally, non-aerosolized VEA and VEA aerosol induce the expression of inflammatory markers such as interleukin (IL)-8 and cyclooxygenase (COX)-2 in an AhR-dependent manner as shown in CRISPR-cas9 AhR-knockout U937-derived human macrophages. These results suggest that VEA is an agonist for AhR and provide new potential mechanisms for lung injury induced by VEA aerosol inhalation via AhR activation in addition to the generation of oxidative stress.

## 1. Introduction

Electronic nicotine delivery systems (ENDS), also known as e-cigarettes, are devices that produce aerosol by heating liquid with or without nicotine. In 2019, hospitalization of chronic e-cigarette users led to categorization of e-cigarette or vaping associated lung injury (EVALI) that show patterns of acute lung injury, foamy macrophages, pneumocyte and neutrophil infiltration (Aldy et al., 2020). Over half illicit vaping cartridges recovered from EVALI patients contain vitamin E acetate (VEA) and the source of VEA is likely to be commercial cannabis-oil diluents (Duffy et al., 2020). Furthermore, VEA was detected in bronchoalveolar lavage fluid from over 90% of EVALI patients, suggesting it to be a culprit in causing EVALI (Blount et al., 2020).

Studies have highlighted adverse effects of exposure to aerosolized VEA. VEA aerosol generated from e-cigarette devices induces cell death and monocyte and neutrophil attracting chemokines such as MCP-1 and IL-8 in human alveolar type II cells in a dose-dependent pattern (Matsumoto et al., 2020). An acute exposure to VEA aerosol in C57BL6 mice results in lung injury characterized by neutrophilic inflammation, lymphocyte infiltration and increase in pro-inflammatory cytokine IL-6, neutrophilic chemokine KC and IL-17 (unpublished results, Bhat et al., 2020).

Although injury related to VEA aerosol exposure has been identified, mechanisms of VEA causing such injury are not well understood. Vitamin E acetate, is a synthetic form of vitamin E. VEA has been widely used in cosmetic products, with claims for improved wound healing and reduced scar tissue (Panin et al., 2004; Thiele et al., 2005). However, more recent studies are showing that there is insufficient evidence for its beneficial effects (Sidgwick et al., 2015; Tanaydin et al., 2016) Thermal degradation of VEA in e-cigarette devices has been shown to generate reactive oxygen species (ROS) as well as carbonyl chemicals that are carcinogens or irritants (Li et al., 2022). Among these compounds, aldehydes such as formaldehyde and acetaldehyde can promote oxidative stress by depleting glutathione due to detoxification (Bernardini et al., 2022). Therefore, oxidative stress may be a main cause of lung injury observed in animals that were exposed to VEA aerosol. In addition to oxidative stress, activation of aryl hydrocarbon receptors (AhR) from other environmental toxicants has also been shown to promote lung inflammation. For instance, activation of AhR by cigarette smoke amplifies Th17 responses including neutrophilic inflammation (Kim et al., 2019).

In this study we focused on the signaling pathways mediating the induction of inflammatory marker genes and the generation of oxidative stress induced after exposure to VEA aerosol in macrophages. The capacity of VEA to activate AhR is an unexpected discovery and further investigations on this AhR agonist may provide more insights on VEA aerosol-induced lung injury and its impact in dermal exposure.

## 2. Methods

### 2.1. Treatment of VEA aerosol extract and non-aerosolized VEA

VEA aerosol was generated by a third-generation e-cigarette device, DNA 75 reference mod for research purposes (Evolv, Hudson, OH) using α-tocopherol acetate (≥ 96% HPLC, Sigma Aldrich, Saint Louis, MO) and device settings of 287.8 °C (550 °F) for temperature and 55 W for power. VEA aerosol was generated by controlled actuation of the device using ‘e-scribe’ software. One ‘puff’ in this study is defined by continuous actuation of the e-cigarette device for 3 s. The aerosol generated from the DNA 75 device used in this study is well-characterized, with an aerosol mass of 2.7 ± 0.3 mg/puff and 55–60 ml/puff when sampled at a flow rate of 1.1–1.2 l (Li et al., 2022). Aerosol was collected on hydrophobic polytetrafluoroethylene (PTFE) membranes (Sigma Aldrich, Saint Louis, MO) via vacuum. Mass of the aerosol was determined by subtracting the post-collection mass of the membrane by its pre-collection mass. Aerosol was extracted by mixing cell culture media with the membrane, followed by vortexing and 30 min of sonication. 20 puffs from the device yielded 70 mg deposit on the membrane. Aerosol-conditioned media was normalized as 100% for 7 mg/ml using 10 ml RPMI culture media. The prepared 7 mg/ml conditioned media was annotated in molar equivalent concentration in VEA (150 μM, 472.74 g/mol). Non-aerosolized VEA was diluted in dimethyl sulfoxide (DMSO, Sigma Aldrich, Saint Louis, MO) before added to the cell culture. Final concentration of DMSO in the cell culture media was 0.5%. For *HO-1* mRNA quantification, 2,5-di-t-butyl-1,4-benzohydroquinone (tBHQ, Sigma Aldrich, Saint Louis, MO) and hydrogen peroxide were used as the positive controls. Lipopolysaccharide (LPS, Sigma Aldrich, Saint Louis, MO) was used as positive control and co-stimulation at a concentration of 100 ng/ml.

### 2.2. Cell culture

The U937 monocytic cells were acquired from the American Tissue Culture Collection in Manassas, VA and maintained for 2 days in RPMI 1640 medium with 10% fetal bovine serum (Gibco, Thermo Fisher Scientific, Waltham, MA, USA), 100 μg/ml streptomycin supplemented with 4.5 g/l glucose. Cell cultures were maintained at concentrations between 2 × 10^5^ and 2 × 10^6^ cells/ml. For macrophage differentiation, U937 cells were treated with 12–0-tetradecanoyl-phorbol-13-acetate (TPA) at 3 μg/ml and allowed to adhere for 2 days to differentiate into macrophages. After differentiation and change into fresh medium macrophages were exposed to the conditioned media for the indicated time points. After exposure, total RNA was extracted and purified using RNA extraction kit (Zymo Research, Irvine, CA).

### 2.3. Generation of CRISPR/Cas9 AhR mutants of U937 cells

CRISPR/Cas9 AhR mutants of U937 cells were generated as described for MCF-7 cells (Vogel et al., 2021). In brief, the CRISPR design tool CHOPCHOP (http://chopchop.cbu.uib.no/ (accessed on 21 March 2023)) was used to design gRNA targeting AhR exon 2 (5′-AAGTCGGTCTCTATGCCGCTTGG-3′). The gRNA was cloned into a PX458 plasmid (Addgene 48,138). U937 cells were transfected with nuclease plasmids in an antibiotic-free medium using FuGENE HD (Roche), according to the manufacturer’s protocol. Cells were sorted (FACS) and plated as single cells in a 96-well plate after 48 h. Clones were genotyped using high-resolution melt analysis and SANGER sequencing. AhR knockout was also confirmed using a DNA/RNA Shield^™^ kit (Zymo Research, Irvine, CA, USA).

### 2.4. Gene expression analysis

Extracted RNA from differentiated macrophages was converted to cDNA using a high-capacity cDNA Reverse Transcription Kit (Applied Biosystem, Waltham, MA). Detection of β-actin as housekeeping gene and differentially expressed target genes was performed with a Light-Cycler LC480 Instrument (Roche Diagnostics, Indianapolis, IN, USA) using the Fast SYBR Green Master Mix (Applied Biosystems, Waltham, MA) according to the manufacturer’s instructions. The primers for each gene were designed based on the respective cDNA or mRNA sequences using OLIGO primer analysis software provided by Steve Rozen and the Whitehead Institute/Massachusetts Institute of Technology Center for Genome Research so that the targets were 100–200 bp in length. To confirm the amplification specificity, the PCR products were subjected to melting curve analysis. The AhR ligand 2,3,7,8-tetrachrolodibenzo-p-dioxin (TCDD) and the TLR4 ligand lipopolysaccharide (LPS) served as positive controls. Gene expression was quantified using the ΔΔ-Ct method and normalized by β-actin (*ACTB*). The primer sequences used in this study are listed in Table 1.

### 2.5. Nrf2 luciferase assay

The HepG2 cells (ATCC HB-8065, Manassas, VA) were maintained in DMEM (Thermo Fisher Scientific, Waltham, MA). The cell culture medium contained 10% fetal bovine serum (FBS) and 100 units of penicillin and 100 μg/ml streptomycin. The Nrf2 luciferase reporter construct and luciferase assay reagents were purchased from Promega (Madison, MI, USA). The luciferase reporter constructs were amplified and purified with a Zymo PURE-Endo Zero plasmid isolation kit (Zymo Research, Irvine, CA, USA). Cells were seeded in 24-well plates and transfected using jetPrime (PolyTransfection; Qbiogene, Irvine, CA, USA). The Nrf2 promoter constructs were suspended in 50 μl of JetPrime reagent and transfection of cells was allowed to proceed for 16 h. Transfected cells were treated with aerosolized VEA (in equivalent molar concentration of VEA) and non-aerosolized VEA for 4 h. Hydrogen peroxide was used as positive controls. A luminometer (Berthold Lumat LB 9501/16, Pittsburg, PA) and 20 μl of cell lysate were used to measure chemiluminescence. Results were expressed as relative light units (RLU).

### 2.6. DRE luciferase assay

To evaluate whether VEA or VEA aerosol can activate AhR, HepG2 cells (ATCC HB-8065, Manassas, VA) — a human liver cell line known for high transfection efficiency and suitability for detecting AhR activation by various ligands — were used. The HepG2 cells were then stably transfected with the PTX.DIR plasmid, which carries a dioxin response element (DRE)-driven luciferase reporter construct, as previously described and kindly provided by Berghard et.al (Berghard et al., 1993). PTX.DIR consists of a xenobiotic response element sequence extending from nucleotides −1026 to −999 relative to the transcription start site of the human *CYP1A1* gene and inserted in a vector containing the herpes simplex virus thymidine kinase promoter and the luciferase reporter gene. The stable transfected HepG2 cells were seeded onto 24-well plates, at a density of 1.2 × 10^4^ cells/well. A VEA aerosol-conditioned media was prepared to match molar concentration in non-aerosolized VEA. Exposures were performed in triplicate for cells treated with VEA or VEA aerosol at 37 °C for 4 h. TCDD was used as a positive control for the DRE activity. Chemiluminescence was measured following the procedure as previously described in Nrf2 luciferase assay.

### 2.7. Intracellular ROS detection

Intracellular oxidative stress was measured using the DCFH-DA assay as previously described (Sciullo et al. 2010). DCFH-DA (Sigma–Aldrich) diffuses into cells and is deacetylated to non-fluorescent DCFH, which is oxidized by ROS to fluorescent DCF. A 2 mM stock solution was prepared in methanol and diluted 1:100 in PBS (final concentration, 20 μM). U937 wild-type and AhR knockout macrophages were seeded in 96-well plates, loaded with DCFH-DA for 1 h at 37 °C, and treated with VEA for 60 min. Fluorescence was measured at 485/530 nm using a Mithras 940LB reader, and ROS levels were calculated after subtraction of control values.

### 2.8. ELISA

The level of IL-8 protein was determined in conditioned medium of U937-derived macrophages using the human IL-8/CXCL8 Quantikine ELISA kit (R&D Systems) according to the manufacturer’s instructions.

### 2.9. Monocyte-derived macrophage cultures

Leukocyte-enriched buffy coats were obtained from healthy donors through the Stanford Blood Center (Mountain View, CA). CD14^+^ monocytes were isolated and differentiated into monocyte-derived macrophages as previously described for monocyte-derived dendritic cells (Kado et al., 2017). Cells were cultured in RPMI 1640 supplemented with 10% endotoxin-free fetal bovine serum and recombinant human GM-CSF (1000 U/ml) for 7 days. On day 6, cells were treated with TCDD or with aerosolized or non-aerosolized VEA for the indicated time period.

### 2.10. Western blotting analysis

CYP1A1 and COX-2 protein levels in U937-derived macrophages were analyzed by Western blotting. Whole cell lysate protein (25 μg) was separated by 10% SDS–PAGE and transferred onto a polyvinylidene difluoride (PVDF) membrane (Immuno-Blot; Bio-Rad) as previously described (Vogel et al. 2014). CYP1A1 and COX-2 mouse monoclonal antibodies were purchased from Santa Cruz Biotechnology. Protein bands were detected using the SuperSignal West Pico chemiluminescent substrate (Pierce) according to the manufacturer’s instructions.

### 2.11. Statistical analysis

Each group of cell cultures and luciferase assay includes 3 biological replicates from different passage numbers. For qPCR, each biological replicate is obtained from the means of 2 technical replicates. Results were reported as mean ± standard error of the mean. Statistical significance was determined with one-way ANOVA and Tukey’s Test at a threshold of *p* < 0.05 using Prism 10.5.0 (Graphpad, Boston, MA).

## 3. Results

### 3.1. Aerosolized VEA induces oxidative stress and proinflammatory responses in U937 macrophages

**Fig. 1.**
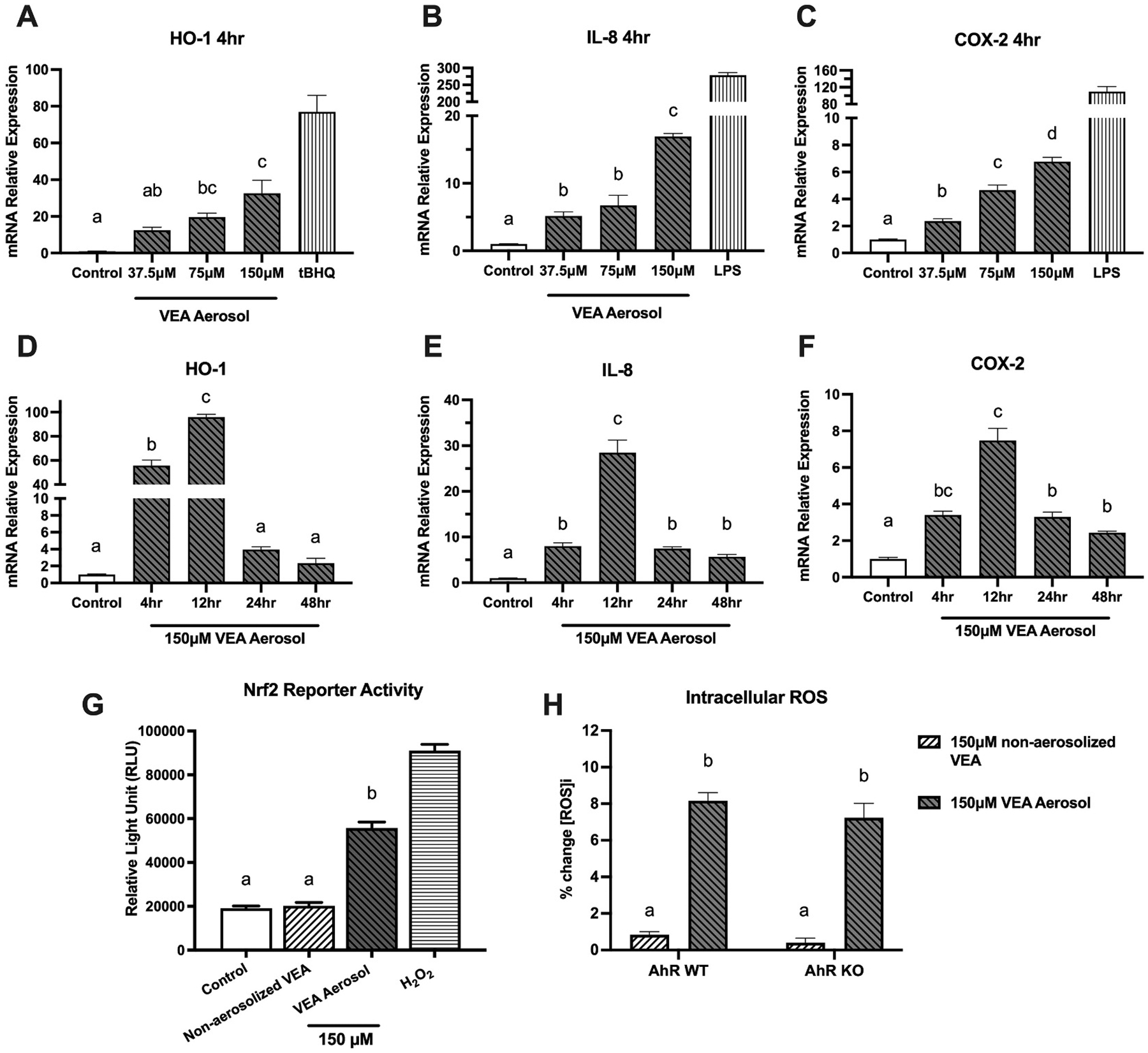
Dose-dependent and time-dependent gene expressions in VEA aerosol-treated macrophages. Dose-dependent mRNA expression of HO-1 (A), IL-8 (B), and COX-2 (C) in U937 macrophages after a 4-h treatment of VEA aerosol extract. Time-dependent expression of HO-1 (D), IL-8 (E), and COX-2 (F) using an undiluted aerosol extract at different time points. G) Nrf2 luciferase activity of 150 μM VEA aerosol-conditioned media and 150 μM non-aerosolized VEA. Hydrogen peroxide (H_2_O_2_) and tBHQ were used as positive controls. Results are expressed in relative light units (RLU), H) Intracellular level of ROS in U937-derived macrophages treated with 150 μM aerosol-conditioned media and 150 μM non-aerosolized VEA for 1 h. Means with different letters are significantly different from each other (Tukey’s HSD, *p* < 0.05).

We first investigated the potential of VEA aerosol to induce oxidative stress and proinflammatory chemokine IL-8, and COX-2. VEA aerosol induced a dose-dependent increase in HO-1, IL-8, and COX-2 expression following a 4-h treatment (Fig. 1A & 1B & C). These expressions peaked following a 12-h treatment and declined after 24 h (Fig. 1D & 1E & F). HO-1 is regulated through the Nrf2 pathway and an antioxidant response element (ARE) sequence on its regulatory promoter region (Campbell et al., 2021). We hypothesized that exposure to VEA aerosols activates the ROS-sensitive transcription factor Nrf2. To test this hypothesis, we measured Nrf2 activation of the VEA aerosol and compared it to non-aerosolized VEA in equivalent molar concentration. Nrf2 activity significantly increased by approximately 2.8-fold in the 150 μМ aerosol-conditioned media relative to the control whereas non-aerosolized VEA had no effect on Nrf2 activity (Fig. 1G). As expected, hydrogen peroxide significantly induced the activity of Nrf2. Next we measured the effects of VEA and VEA aerosol on the generation of intracellular ROS. Treatment with VEA aerosol at 150 μМ for 1 h in the human monocyte-derived U937 macrophages results in significant increase in intracellular ROS compared to the non-aerosolized VEA counterpart (Fig. 1H). Furthermore, The results depicted in Fig. 1H also support that the generation of ROS induced by VEA aerosol does not require the function of AhR since VEA aerosol induced ROS production in AhR knockout U937-derived macrophages similar to wildtype U937-derived macrophages expressing a functional AhR.

### 3.2. Role of AhR in VEA aerosol-induced CYP1A1 and HO-1 mRNA expression

**Fig. 2.**
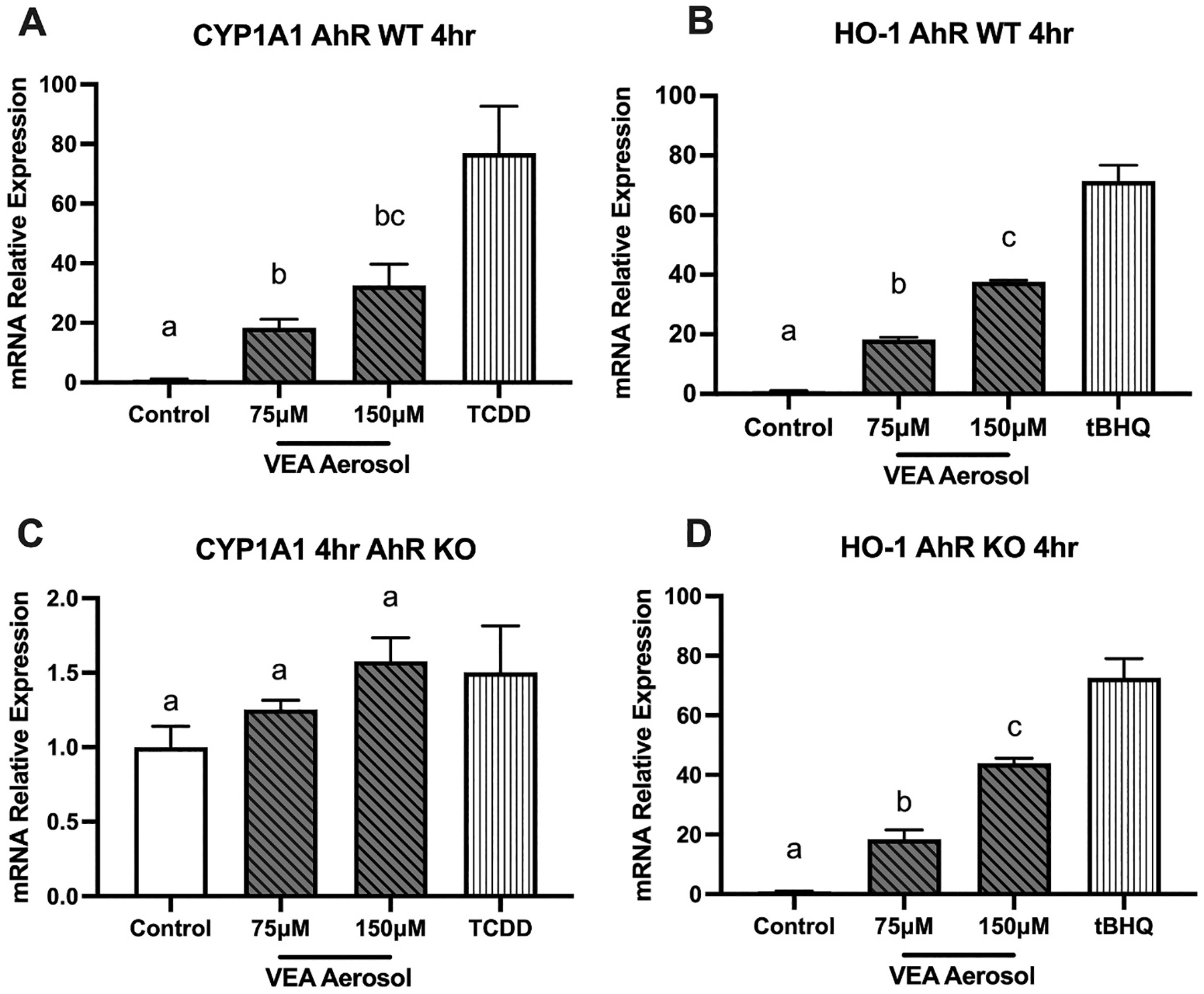
Gene expressions in VEA aerosol-treated macrophages. CYP1A1 and HO-1 mRNA expression in AhR wildtype (WT) (A&B) and knockout (KO) U937 macrophages (C&D) treated with 150 μM VEA aerosol-conditioned media. Means with different letters are significantly different from each other (Tukey’s HSD, *p* < 0.05).

In addition to oxidative stress, we screened AhR activity in response to VEA aerosol. VEA aerosol-conditioned media induced CYP1A1 and HO-1 mRNA expression at 75 μM and 150 μM concentrations in a dose-dependent manner (Fig. 2A & 2B). The aerosol extract at 75 μM and 150 μM concentrations induced approximately 8-fold and 30-fold increase in CYP1A1 mRNA, respectively (Fig. 2A). The treatment with aerosol extract had no effect on CYP1A1 in AhR knockout macrophages (Fig. 2C). Interestingly, VEA aerosol induced similar levels of HO-1 mRNA expression in the wildtype and AhR knockout macrophages (Fig. 2B & 2D).

### 3.3. Non-aerosolized VEA activates AhR and induces CYP1A1, IL-8, and COX-2 mRNA and protein expression

**Fig. 3.**
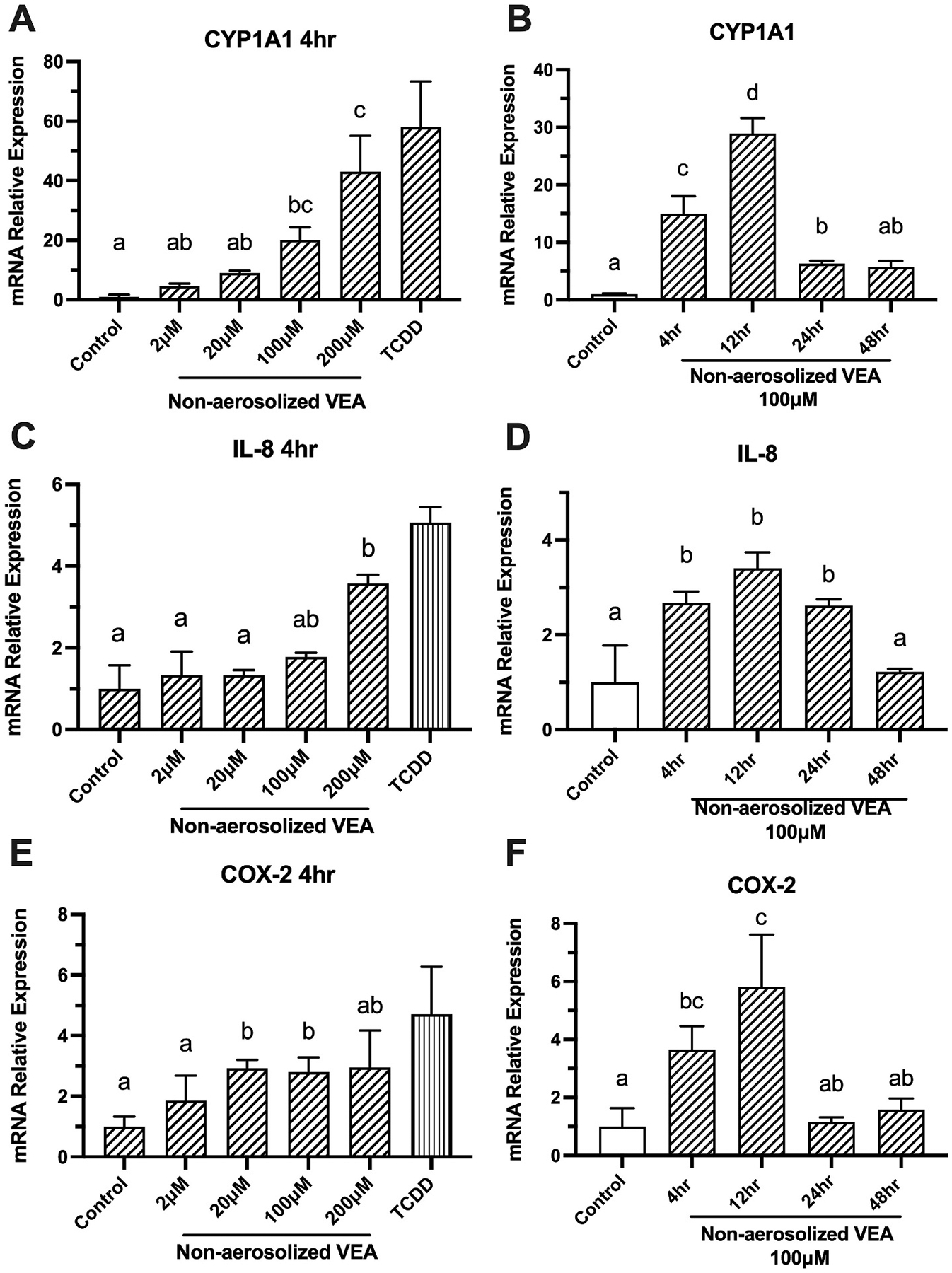
Gene expressions of non-aerosolized VEA treated macrophages. A) CYP1A1, B) IL-8 and C) COX-2 mRNA expression in U937 macrophages after treatment for 4 h with different concentrations of non-aerosolized VEA. Time-dependent expression of D) CYP1A1, E) IL-8 and F) COX-2 mRNA expression after treatment of non-aerosolized VEA. Means with different letters are significantly different from each other (Tukey’s HSD, *p* < 0.05).

After observing AhR activities of the VEA aerosol, we tested the potency of non-aerosolized VEA on AhR activities since it is the major component of the aerosol. Non-aerosolized VEA induced an increase in both CYP1A1 and IL-8 expression following a 4-h exposure from 2 μM to 200 μM. At 20 μM, 100 μM and 200 μM, VEA induced approximately 10-, 20- and 40- fold CYP1A1 expression (Fig. 3A). The increase in CYP1A1 expression was significant at 100 and 200 μM. At 200 μM, VEA induced a significant 3-fold increase in IL-8 mRNA and such increase is comparable to well-established AhR ligand TCDD at 1 nM (Fig. 3C). Starting from 20 μM, non-aerosolized VEA induced a significant 3-fold expression of COX-2 (Fig. 3E).

Following the dose responses, we investigated the time-dependent AhR activity by the treatment of VEA. At 100 μМ, non-aerosolized VEA induced a significant 15-fold induction of CYP1A1 mRNA at 4-h after treatment and the induction peaked after 12-h at approximately 30-fold (Fig. 3B). After 24 or 48 h, CYP1A1 activity declined and approach baseline levels. IL-8 expression also significantly peaked after 12 h of treatment at approximately 4-fold and gradually declined after 24 and 48 h (Fig. 3D). In a similar fashion, COX-2 expression significantly peaked after 12 h at about 6-fold above control and declined after 24 and 48 h (Fig. 3F).

**Fig. 4.**
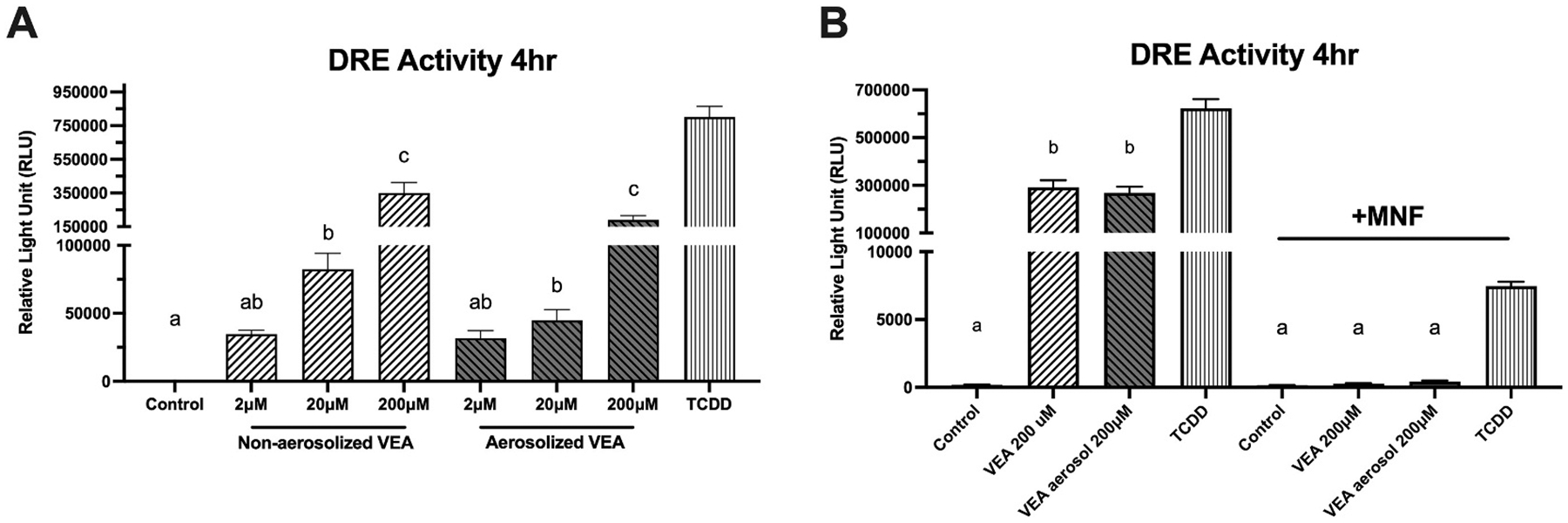
A) DRE luciferase activity of different concentrations of non-aerosolized VEA and VEA aerosol. B) DRE luciferase activity of non-aerosolized VEA and VEA aerosol in presence of an AhR antagonist MNF. Concentrations of aerosol were normalized to match non-aerosolized VEA based on mass. Means with different letters are significantly different from each other (Tukey’s HSD, p < 0.05).

To compare AhR activity of the VEA aerosol and non-aerosolized VEA, we measured DRE activity using an in vitro luciferase reporter assay. The concentration of the aerosol was normalized to molarity of non-aerosolized VEA using mass. Both non-aerosolized VEA and VEA aerosol showed comparable AhR activity at same concentrations. Even at 2 μM, both VEA aerosol and non-aerosolized VEA upregulated AhR activities (Fig. 4A). Additionally, we tested the AhR antagonist 3′-methoxy-4’nitroflavone (MNF) on VEA aerosol and non-aerosolized VEA-induced AhR activity. The results showed that MNF completely blocks the AhR activity induced by VEA aerosol and non-aerosolized VEA (Fig. 4B), indicating that both VEA aerosol and non-aerosolized mediate the induction of CYP1A1 via the AhR pathway.

**Fig. 5.**
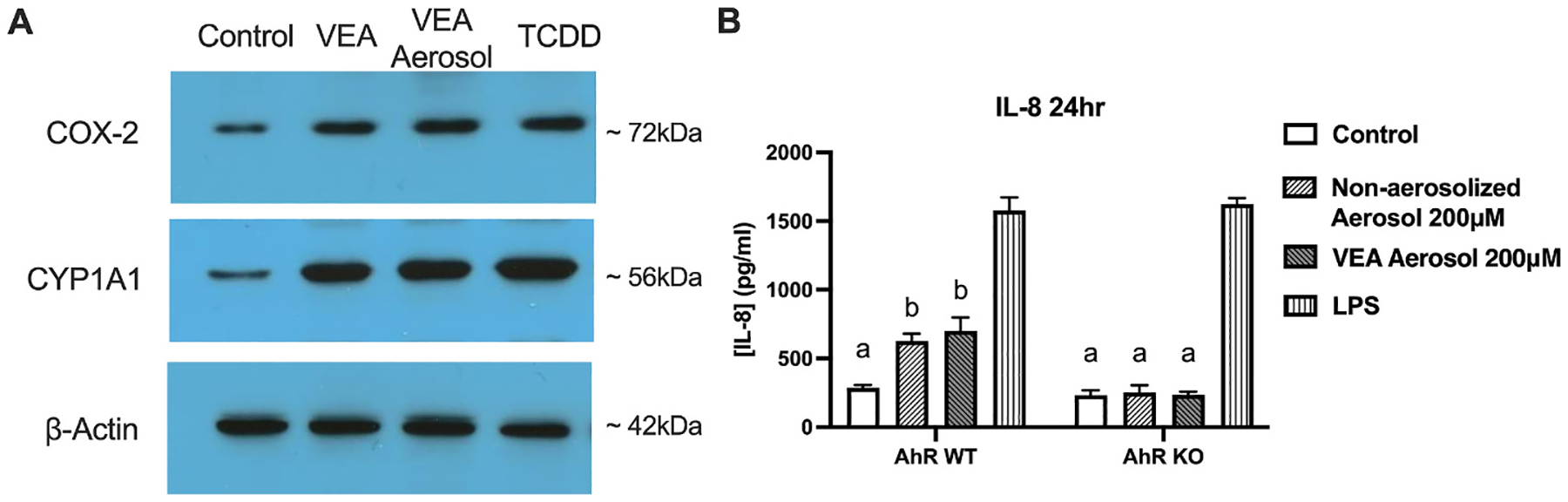
VEA aerosol and non-aerosolized VEA induce CYP1A1, COX-2 and IL-8 protein expression. A) Western blot analysis of CYP1A1 and COX-2 in whole cell lysate of U937 macrophages after 24 h treatment with 200 μM non-aerosolized VEA and 200 μМ VEA aerosol. B) Analysis of IL-8 protein in supernatant of U937 macrophages after 24 h treatment with 200 μM non-aerosolized VEA and 200 μМ VEA aerosol. 1 nM TCDD was used as a positive control. Means with different letters are significantly different from each other (Tukey’s HSD, p < 0.05).

**Fig. 6.**
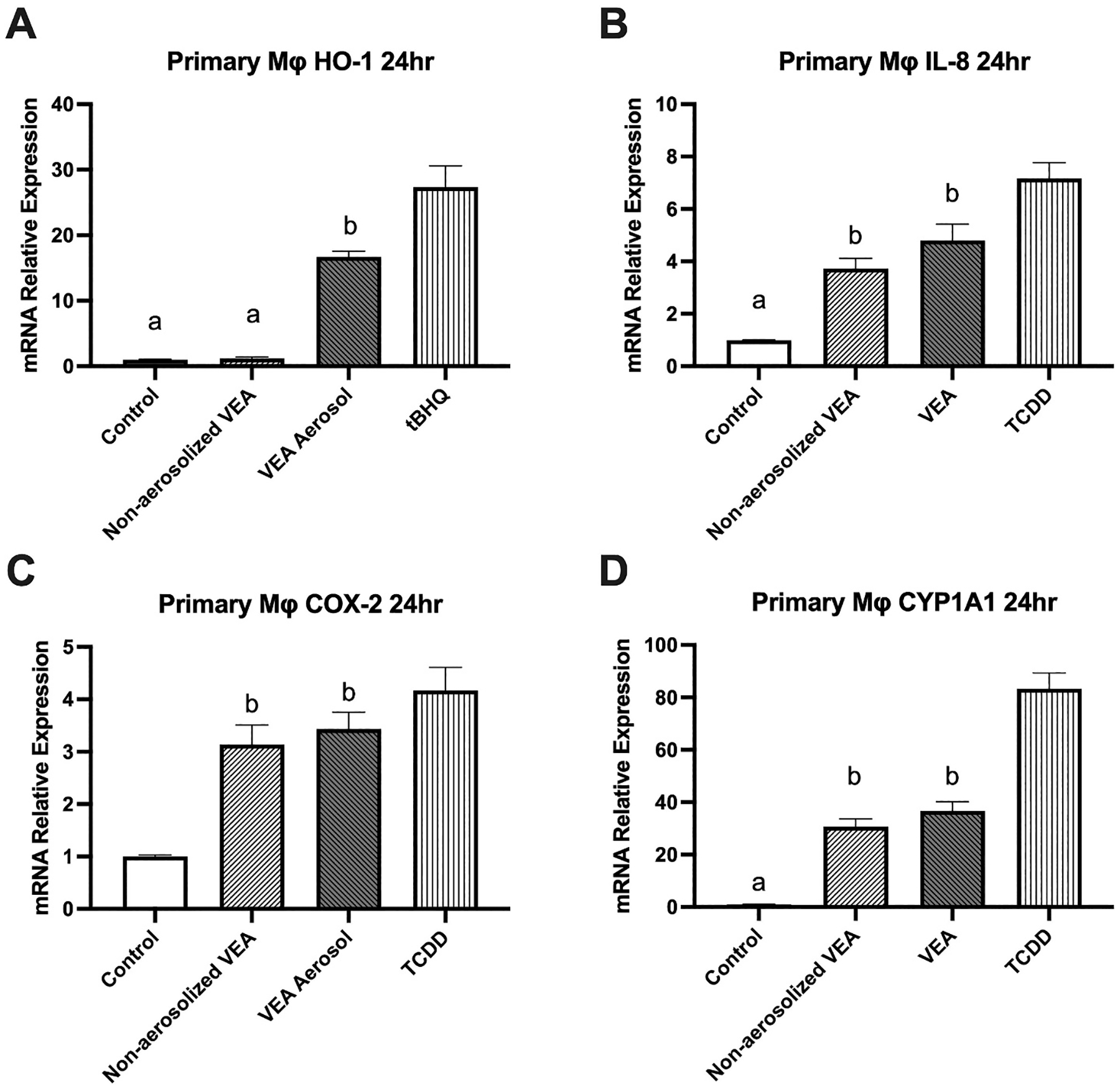
mRNA expressions of HO-1 (A), IL-8 (B), COX-2 (C) and CYP1A1 (D) in primary human macrophages. Macrophages were treated with 200 μM non-aerosolized VEA and 200 μM VEA aerosol. Cells were treated for 24 h and mRNA expression was analyzed using qPCR and normalized to β-actin. Means with different letters are significantly different from each other (Tukey’s HSD, p < 0.05).

Western blot analysis confirmed an elevated protein level of CYP1A1 and COX-2 in U937-derived macrophages after treatment with VEA aerosol and non-aerosolized VEA (Fig. 5A). Additionally, we measured the production of IL-8 protein after treatment with VEA using ELISA. The results show a significant increase of IL-8 protein in the supernatant of U937-derived macrophages after treatment with VEA aerosol and non-aerosolized VEA for 24 h (Fig. 5B). Furthermore, we investigated the effect of non-aerosolized and VEA aerosol on the mRNA expression of CYP1A1, COX-2, IL-8 and HO-1 in human primary macrophages (Fig. 6A–D). Both VEA aerosol and non-aerosolized VEA induced the expression of CYP1A1, IL-8, and COX-2 in primary human macrophages of healthy donors. However, only aerosolized VEA induced the expression of HO-1, confirming data from U937-derived macrophages.

### 3.4. Non-aerosolized VEA modulates expression of proinflammatory cytokines in LPS-stimulated macrophages

**Fig. 7.**
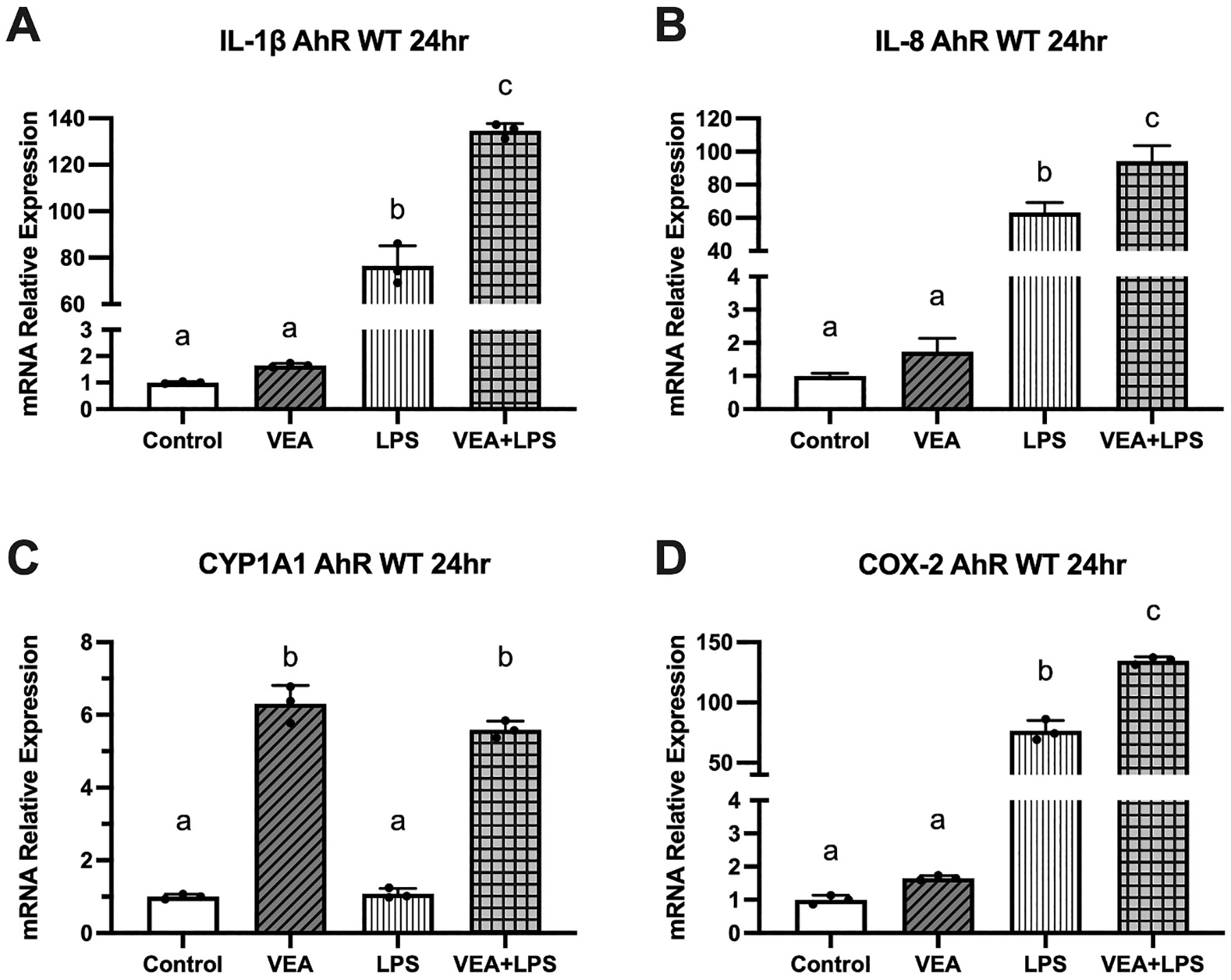
Gene expressions of VEA and LPS co-stimulation in AhR wildtype U937 macrophages. IL1-β (A), IL-8 (B), COX-2 (C) and CYP1A1 (D) expression after 24 h treatment with 100 μM non-aerosolized VEA and 100 ng/ml LPS. Means with different letters are significantly different from each other (Tukey’s HSD, p < 0.05).

**Fig. 8.**
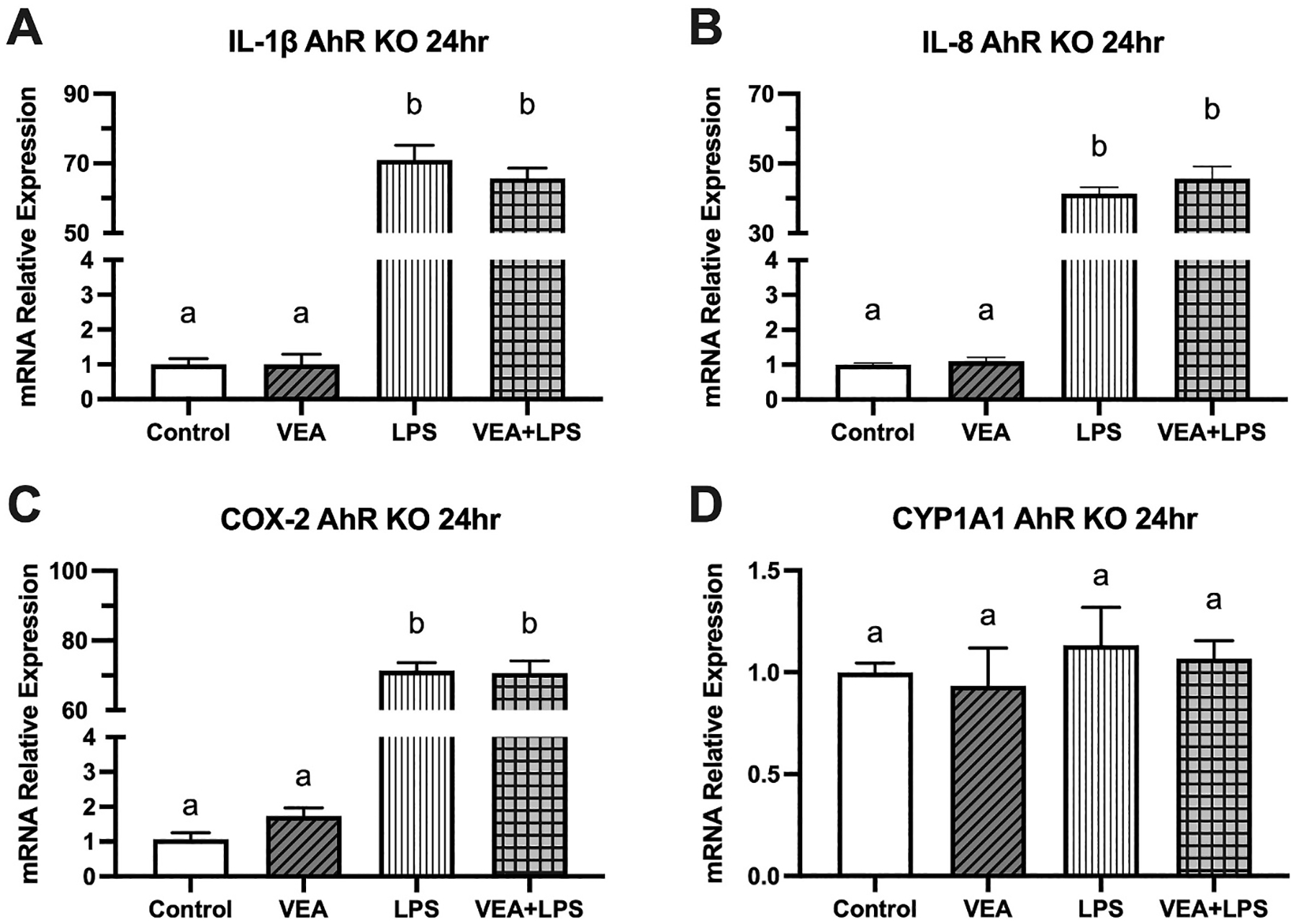
Gene expressions of VEA and LPS co-stimulation in AhR knockout U937 macrophages. IL1-β (A), IL-8 (B), COX-2 (C) and CYP1A1 (D) expression after 24 h treatment with 100 μM non-aerosolized VEA and 100 ng/ml LPS. Means with different letters are significantly different from each other (Tukey’s HSD, p < 0.05).

We also tested the effect of non-aerosolized VEA on inflammatory responses in LPS-activated macrophages. LPS significantly increased the expression of IL-1β, IL-8 and COX-2 after 24 h treatments. Simultaneous treatment of non-aerosolized VEA and LPS for 24 h further increased expression of IL-1β, IL-8 and COX-2 by approximately 50% relative to LPS treatment alone (Fig. 7A,B & C). LPS did not significantly alter expression of CYP1A1 induced by VEA (Fig. 7D). Additionally, we tested the effect of VEA on LPS-induced cytokines and COX-2 in AhR knockout macrophages. VEA did not induce CYP1A1 expression or enhance the LPS-induced expression of IL-1β, IL-8, and COX-2 in AhR knockout macrophages (Fig. 8A–D).

## 4. Discussion

Lipid-laden macrophages from EVALI patients and foamy, vacuolated macrophages identified in animal studies indicate significant uptake of VEA by macrophages (Marrocco et al., 2022). Thus, a macrophage model renders relevance to studying adverse effects of the exposure to VEA aerosol. The increased generation of ROS, the induction of HO-1 and Nrf2 activity induced by VEA aerosol indicates that exposure to VEA aerosol generates reactive oxygen species and thus elicits oxidative stress in U937 macrophages. The selection of the U937-derived macrophages to test VEA aerosol was based on our previous studies testing the responsiveness of U937-derived macrophages to oxidized lipids and inflammatory stimuli (Vogel et al., 2004). Recent reports including our own studies have shown that U937-derived macrophages respond to oxidized low-density lipoprotein and cholesterol mediating formation of cholesterol-rich foam cells associated with the release of pro-inflammatory cytokines (Vogel et al., 2004; Vogel et al., 2005). Furthermore, we found similar responses to inflammatory stimuli in primary human monocyte-derived dendritic cells compared to U937-derived dendritic cells suggesting that the human U937-derived macrophages serve as a suitable model to study the response to lipids and inflammatory stimuli (Kado et al., 2017). For the current study, the target genes IL-8, COX-2, and CYP1A1 were chosen for analysis since they belong to a group of genes which have been found to be affected after exposure to particulate matter and their organic extracts derived from combustion processes (Vogel et al., 2005).

Here, we found HO-1 mRNA expression and ROS generation in AhR knockout macrophages indicating that oxidative stress caused by VEA aerosol is independent to AhR activation. The chemical in the aerosol that induced ROS and HO-1 expression has not been identified in this study but could be mediated by duroquinone, which has been characterized in VEA aerosol and causes oxidative stress and cytotoxicity in BEAS-2B cells (Canchola et al., 2022). In addition to induction of oxidative stress, the capacity of VEA aerosol extract to induce AhR activation by VEA may also elucidate pronounced neutrophilic inflammation identified in in vivo exposures to the VEA aerosol. The capacity of both non-aerosolized VEA and VEA aerosol to induce CYP1A1 expression and DRE activity in the luciferase reporter assay clearly indicates that VEA is an agonist for AhR. Furthermore, data from U937-derived AhR knockout macrophages shows that the induction of CYP1A1 is AhR-dependent.

Interestingly, in a recent study, alpha-tocopherylquinone, a quinone-structured oxidation product of vitamin E, has been found to activate AhR and Nrf2 in Caco-2 cells to enhance the basal intestinal epithelial tight junction’s barrier (Ganapathy et al., 2023). This study and the data of the current study indicate that the oxidation product of vitamin E (alpha-tocopherylquinone) and alpha-tocopherol acetate or VEA, a synthetic derivative of vitamin E (alpha-tocopherol acetate or VEA) may function as AhR agonists and regulate AhR as well as Nrf2 activity. AhR agonists such as benzo(*a*)pyrene have been shown to promote lung inflammation via induction of neutrophilic cytokine IL-8 (Podechard et al., 2008). Agonist such as 6-formylindolo[3,2-*b*]carbazole (FICZ) can also exacerbate inflammation by inducing COX-2 and differentiation of IL-17А secreting cells including T-helper 17 (Th17) cell and group 3 innate lymphoid cells (Enweasor et al., 2021; Fritsche et al., 2007; Ivanov et al., 2006; Quintana et al., 2008). IL-17 A secreting cells play a major role in neutrophil-mediated pathogenesis since it can activate nonimmune cells such as epithelial cells to induce pro-inflammatory cytokines including G-CSF and IL-8, thereby promoting maturation and recruitment of neutrophils (Korn et al., 2009). In the presence of oxidative stress induced by degradation products of VEA, AhR agonism may exacerbate neutrophilic inflammation via activation of IL-8 and COX-2.

AhR activation in macrophages can promote either proinflammatory or anti-inflammatory cytokines depending on the ligand. Exposure to environmental toxicants such as particulate matter 2.5(PM_2.5_) led to increased expression of IL-1β and IL-8 (Malany et al., 2024). In a murine model, exposure to PM_2.5_ during allergen sensitization exacerbates asthmatic responses when challenged by house dust mite allergen (Yuan et al., 2022). The capacity of VEA to promote expression of IL-1β, IL-8 and COX-2 expression in an LPS co-stimulation model suggests that exposure to VEA can influence the immune responses to infections and may also modulate simultaneous exposure to other environmental toxicants.

This study has some limitations. Aerosol collected on PTFE membranes lacks volatile chemicals in the extracted aerosol. Thus, capacity of VEA aerosol to induce oxidative stress could be underestimated since cells were not exposed to volatile organic chemicals such as formaldehyde and acetaldehyde formed during thermal degradation of VEA.

Together, AhR and Nrf2 activity can be induced by VEA as well as alpha-tocopherylquinone, suggesting that derivatives of Vitamin E can modulate the immune system in addition to their antioxidative functions (Ganapathy et al., 2023). These findings indicate that metabolites of Vitamin E may play a dual role as antioxidants and as signaling molecules that influence cellular stress responses. The results of this study also show that both, VEA and VEA aerosol activate AhR signaling and induce CYP1A1, IL-8 and COX-2 in an AhR-dependent manner. However, only VEA aerosol generates ROS and activates Nrf2 independent of AhR. The results support that activation of AhR and downstream induction of CYP1A1, COX-2 and IL-8 by VEA do not require the generation of ROS or activation of Nrf2. The biological effects of AhR and Nrf2 activation by Vitamin E derivatives could provide new strategies for the development of non-toxic molecules in clinical applications. However, the potential beneficial effects of Vitamin E derivatives and adverse effects as described for VEA—particularly in the context of inhalation and dermal exposure—merit further investigations. A deeper understanding of their metabolic fate, tissue distribution, and context-specific biological activities is essential to evaluate their therapeutic potential and safety profiles.

## Figures and Tables

**Table 1:** Primer sets used in RT-qPCR.

Gene	Forward (−5′ to 3′-)	Reverse (−5′ to 3′-)
*CYP1A1*	GAGGCCAGAAACTCCGT	CCCAGCTCAGCTCAGTACCT
*HMOX1*	ATGACACCAAGGACCAGAGC	GTCTAAGGACCCATCGGAGA
*IL1*β	GGGCCTCAAGGAAAAGAATC	TTCTGCTTGAGAGGTGCTGA
*IL8*	TAGCAAAATTGAGGCCAAGG	AAACCAAGGCACAGTGGAAC
*ACTB*	CATCCGCAAAGACCGGTACG	CCTGCTTGCTGATCCACATC
*COX2*	GGAACACAACAGAGTATGCG	AAGGGGATGCCAGTGATAGA

Note: *CYP1A1*, cytochrome P450, family 1, subfamily A, polypeptide 1; *HMOX1*, hemeoxygenase-1(HO-1); *IL*, interleukin; *ACTB,* β-actin; *COX2*, cyclooxygenase 2 (COX-2).

## Data Availability

No data was used for the research described in the article.
